# *Escherichia fergusonii* Associated with Pneumonia in a Beef Cow

**DOI:** 10.1155/2013/829532

**Published:** 2013-12-02

**Authors:** Guillermo M. Rimoldi, Robert B. Moeller

**Affiliations:** California Animal Health and Food Safety Laboratory System (CAHFS), Davis and Tulare Branches, 620 West Health Sciences Drive, Davis, CA 95616, USA

## Abstract

An adult Angus cow developed hyperthermia, prostration, and respiratory distress, dying 36 hours after the onset of clinical signs. The main finding during postmortem examination was a severe focally extensive pneumonia. Icterus and a chronic mastitis were also noticed. Histologic examination of the lungs detected fibrinonecrotic pneumonia, with large number of oat cells and intralesional Gram-negative bacterial colonies. Samples from lung lesions were collected, and a pure growth of *Escherichia fergusonii* was obtained. *E. fergusonii* is a member of Enterobacteriaceae, related to *Escherichia coli* and *Salmonella* sp. In veterinary medicine, *E. fergusonii* has been reported in calves and sheep with clinical cases suggestive of salmonellosis; in a horse and a goat with enteritis and septicemia; and in ostriches with fibrinonecrotic typhlitis. To our knowledge, this report represents the first description of *E. fergusonii* associated with an acute pneumonia in cattle.

## 1. Introduction

*Escherichia fergusonii* was described as a new species of the genus Escherichia, family Enterobacteriaceae in 1985 [1]. It is closely related to *E. coli*, with whom it shares many of its biochemical properties and resembles the genus *Salmonella *in the lack of lactose breakdown activity. When first identified, the clinical significance of this organism was unclear. First isolate suggesting that this organism was a pathogen was from a human patient with pancreatic carcinoma and cholangiosepsis, in Switzerland. The organism was detected in the blood, gallbladder fluid, feces, and a superficial abdominal wound [2]. In the veterinary literature, *E. fergusonii* has been associated with a clinical case of acute enteritis, septicemia, and death of an adult horse from Germany [3]. *E. fergusonii* was also isolated from intestine, lung, liver, and kidney from a goat with a history of chronic diarrhea and emaciation, in Canada [4]. In cattle, *E. fergusonii* has been isolated from animals with diarrhea, clinically suggestive of salmonellosis [5]. Birds seem to be also susceptible to *E. fergusonii* infections; this bacterium was isolated in pure growth from lesions in ostriches with severe hemorrhagic diarrhea and fibrinonecrotic typhlitis [6]. Common pulmonary bacterial pathogens in cattle include *Mannheimia haemolytica*, *Pasteurella multocida*, *Histophilus somni, Mycoplasma bovis*, and* Trueperella *(*Arcanobacterium*)* pyogenes*. Young animals are more prone to develop pneumonias caused by one or more of these agents; however, adult animals are also susceptible, particularly when immunocompromised. In this report, *Escherichia fergusonii* was isolated in pure culture from otherwise pneumonic lesions, where one or more of the common bacterial pathogens for cattle were expected to be found.

## 2. Case Description

A 4-year-old Angus cow, pregnant, nursing her 6-month-old heifer, was found down, alert, tachypneic, and with hyperthermia (41.7°C). The herd had been moved to a new irrigated pasture the day before the animal was presented for clinical evaluation. The referring veterinarian treated the cow with IV fluids, Flunixin Meglumine, and Ceftiofur. Despite treatments, the animal was found dead the next day and submitted for postmortem examination together with a blood sample drawn the previous day. The case was described as acute, lasting only 36 hours; other herdmates were not affected.

On necropsy, the animal was in fair postmortem state, good nutritional status, mildly dehydrated, and icteric. A 25 cm. long fetus was present in the uterus (estimated 4-month gestation). Approximately 50% of the lungs were bilaterally consolidated and reddened with interlobular septa accentuation. This was particularly evident in the cranioventral region. The spleen was enlarged (1.5x) with a mottled appearance. The small intestine was gas distended with scant content in the lumen. The cecum had a dark brown runny content, and the spiral colon had dry brown feces. The liver was tan/orange, slightly swollen with a marked reticular pattern. The rest of the organs did not have significant lesions. The packed cell volume was determined in the submitted blood sample and found to be 26%.

**Figure 1:**
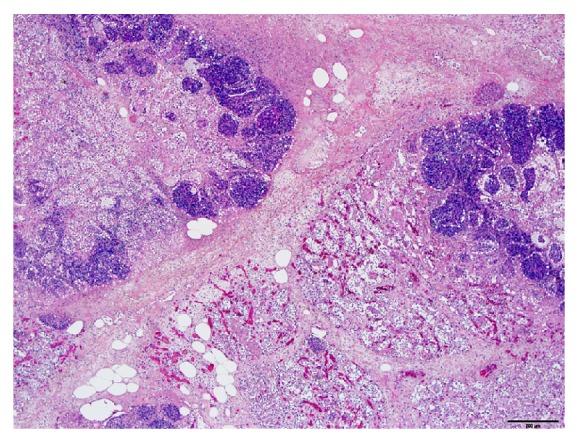
Histological appearance of the affected lung tissue, fibrinous bronchopneumonia with large alveolar necrotic areas filled with neutrophils and debris. Interlobular septa are markedly expanded with fibrin, edema, and lesser numbers of neutrophils. Hematoxylin and eosin, 4x, bar = 200 *μ*m.

**Figure 2:**
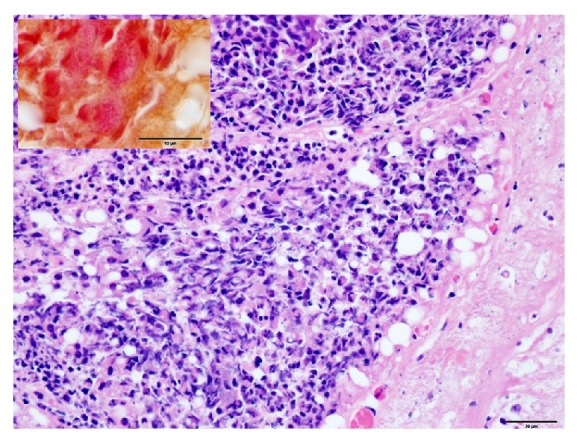
Outermost section of a necrotic area, densely packed neutrophils, and numerous oat cells. Hematoxylin and eosin, 40x, bar = 20 *μ*m. Insert (upper left) displays intralesional, bacterial colonies, Gram-negative coccobacilli. Hucker-Conn Gram stain 100x, bar = 10 *μ*m.

Sections of lung, liver, spleen, kidney, heart, rumen, abomasum, small, intestine, spiral colon, mammary gland, and brain were fixed in 10% buffered formalin, routinely processed for histology and examined. The primary microscopic lesion was identified in the lungs. It consisted of a fibrinonecrotic bronchopneumonia with large, irregular, necrotic alveolar areas, filled with fibrin and surrounded by densely packed neutrophilic infiltrates, including oat cells, suggestive of *Mannheimia* spp. or *Pasteurella* spp. infection (Figure 1). Within the necrotic areas, large numbers of Gram-negative coccobacilli were detected (Figure 2). Lymphatic vessels within interlobular septa were severely distended, filled with fibrin and debris. The liver displayed centrilobular hepatocellular degeneration, with incipient hepatocyte necrosis (suggestive of hypoxic changes), mild histiocytic infiltration in sinusoids, and increased bile in canaliculi. The mammary gland had a chronic-active suppurative mastitis with increased interlobular fibrosis, multifocal acinar necrosis, neutrophilic exudates into the acinar and ductal lumina, and moderate numbers of intralesional Gram-negative coccobacilli. Rare intraerythrocytic parasites consistent with *Anaplasma *sp. were detected with Wolbach-Giemsa staining in the liver and lungs and in a smear from the fresh blood sample stained with Diff-Quick (in >1% of RBC, estimated).

Lungs and liver were cultured on blood-agar and MacConkey-agar plates, incubated for 48 hours, 37°C in aerobic, CO_2_ enriched conditions (7% CO_2_). Culture identification was performed with standard biochemical procedures and confirmed with commercial enteric identification system (API 20 E test by Biomerieux). The single bacterial isolate in pure culture and in large numbers from two lungs and one liver samples was acid/acid (A/A), gas producer, and H_2_S negative on triple sugar iron media (TSI), motile, indole, and ornithine decarboxylation positive on motility-indole-ornithine media (MIO), orthonitrophenyl-*β*-galactoside (ONPG) positive, urea hydrolysis positive and negative for citrate utilization. Based on these findings isolate was identified as *Escherichia fergusonii*. Given the unusual bacteriological results and lung histology suggestive of another, rather than *Escherichia* spp. genus bacteria involved, routinely frozen tissues during necropsy were thawed and recultured. *E. fergusonii* was isolated again from lung, in pure culture and large numbers. On Kirby-Bauer disk diffusion test, using interpretation criteria as with *E. coli* [7], the isolate was resistant to Ceftiofur, Erythromycin, Penicillin, and Ampicillin, and it was sensitive to Neomycin, Streptomycin, Tetracycline, and Florfenicol.

*Anaplasma* infection was confirmed by PCR, test performed on splenic tissue at the Washington Animal Disease Diagnostic Laboratory as previously described [8]. *Leptospira* was ruled out by fluorescence antibody test (FAT) on kidney smears (Leptospira fluorescent antibody conjugate, National Veterinary Services Laboratory). McMasters test detected rare numbers of coccidian oocysts (100/gram). Liver selenium levels were low (0.096 ppm, ref. range 0.25–0.5 ppm), copper, zinc, and iron were within normal limits and mercury, and arsenic, cadmium, and lead were not detected.

## 3. Conclusions

*E. fergusonii* pathogenic capability has been demonstrated as reported in multiple species including humans, several mammalians, and birds [2–6]. Here, *E. fergusonii* appears to be responsible for a severe, acute pneumonia and death in an adult cow. It is possible that stressors such as an *Anaplasma* infection with mild anemia, low selenium levels, and advanced pregnancy while nursing a 6-month-old calf may have contributed to an immunocompromised animal susceptible to this bacterium. While the lesions were histologically suggestive of *Mannheimia/Pasteurella* pneumonia (oat cells formation) and assuming *E. fergusonii* would produce *Escherichia coli*-like lesions (suppurative rather than fibrinonecrotic), recovering *E. fergusonii* in large numbers and pure culture from 3 lung sampling sites (2 on necropsy and 1 reculture) would suggest that this pneumonia was due to the bacterium. It is possible that *Mannheimia/Pasteurella* started the pneumonic process, induced the lesions associated with these bacteria, and then were controlled with antimicrobial treatment. Under this condition, if *E. fergusonii* was present it was then able to multiply and appear as the agent responsible for the proceeding severe, acute pneumonia leading to death. It is highly unusual for a single dose of an antimicrobial product to clear a pathogen such as *Mannheimia/Pasteurella* when death occurs soon (less than 24 hours) after the initiation of treatment. If this scenario happened, *E. fergusonii* should still be considered an opportunistic but potentially a severe pulmonary pathogen for cattle. Postmortem contamination was considered but if *E. fergusonii *was a relatively common contaminant, it would be frequently detected in animals submitted for necropsy with some autolysis and often in mixed cultures. *E. fergusonii *is rarely isolated from necropsy samples in our bacteriology laboratory, never in large numbers and pure culture. Isolation from liver is suggestive of a potential bacteremic spread when disease was advanced or terminal. We believe that *E. fergusonii *is an organism that under certain circumstances might act as a lung pathogen in cattle. To our knowledge, this is the first case where *E. fergusonii* is reported as a pulmonary pathogen in cattle.
